# Emergent dynamics in a robotic model based on the *Caenorhabditis elegans* connectome

**DOI:** 10.3389/fnbot.2022.1041410

**Published:** 2023-01-09

**Authors:** Carlos E. Valencia Urbina, Sergio A. Cannas, Pablo M. Gleiser

**Affiliations:** ^1^Medical Physics Department, Centro Atómico Bariloche, Instituto Balseiro, Universidad Nacional de Cuyo, Río Negro, Argentina; ^2^Facultad de Matemática, Astronomía, Física y Computación, Universidad Nacional de Córdoba, Instituto de Física Enrique Gaviola (IFEG-CONICET), Ciudad Universitaria, Córdoba, Argentina; ^3^Laboratorio de Neurociencia de Sistemas Complejos, Departamento de Ciencias de la Vida, Instituto Tecnològico de Buenos Aires (ITBA), Buenos Aires, Argentina

**Keywords:** self-organized systems, synchronization, connectome, *C. elegans*, robot

## Abstract

We analyze the neural dynamics and their relation with the emergent actions of a robotic vehicle that is controlled by a neural network numerical simulation based on the nervous system of the nematode *Caenorhabditis elegans*. The robot interacts with the environment through a sensor that transmits the information to sensory neurons, while motor neurons outputs are connected to wheels. This is enough to allow emergent robot actions in complex environments, such as avoiding collisions with obstacles. Working with robotic models makes it possible to simultaneously keep track of the dynamics of all the neurons and also register the actions of the robot in the environment in real time, while avoiding the complex technicalities of simulating a real environment. This allowed us to identify several relevant features of the neural dynamics associated with the emergent actions of the robot, some of which have already been observed in biological worms. These results suggest that some basic aspects of behaviors observed in living beings are determined by the underlying structure of the associated neural network.

## 1. Introduction

Understanding how the nervous system of living organisms encodes, organizes and sequences behaviors is one of the fundamental questions in biology and science in general (Branson and Freeman, 2015). The main challenge resides in the ability to monitor whole, or almost whole neural systems while registering activity (Anderson and Perona, 2014; Datta et al., 2019). With this goal in mind, many studies have focused on model animals with small nervous systems, such as the fruit fly *Drosophila melanogaster* (Robie et al., 2017), the zebrafish *Danio rerio* (Ahrens et al., 2013; Cong et al., 2017; Kim et al., 2017; Symvoulidis et al., 2017), and the worm *Caenorhabditis elegans* (*C. elegans*) (Corsi et al., 2015; Kato et al., 2015; Kaplan et al., 2020).

The nematode *C. elegans* stands out in neuroscience studies as the first animal whose complete connectome has been mapped (White et al., 1986). Using serial electron microscopy synapse-level neural maps have been constructed both for adult male and hermaphrodite (White et al., 1986; Cook et al., 2019). By adding the sizes of the synaptic connections between cell pairs, and assuming that average synapse size is larger for stronger connections than for weak ones, the map can be represented as an adjacency matrix with weights that quantify the amount of physical connectivity between pairs. This allows for the description of the nervous system as a weighted graph.

The abstraction of a neural system into a set of nodes and weighted edges allows for the development of a theoretical framework to study the general organizing principles of the neural structures. This approach has proven to be successful (Sporns et al., 2004; Sporns, 2010; Fornito et al., 2016; Van den Heuvel et al., 2016), revealing many non trivial topological features, that are shared by nervous systems across species, such as network motifs (Sporns et al., 2004), community structures (Sohn et al., 2011), rich clubs (Towlson et al., 2013), and small world network structure (Watts and Strogatz, 1998; Varshney et al., 2011). Also, it establishes the first step in the study of the relation between the network structure and function, that is, on the dynamical processes that can run on these structures. For example, the small world network structure is characterized by a high clustering and a short distance between nodes. This allows for the coexistence of a functional segregation in well defined regions while also allowing for a fast transfer of information consolidating global integration into coherent states (Sporns et al., 2004).

The possibility of advancing beyond structural analysis, incorporating experimental information on neural dynamics has also been possible in *C. elegans*, thanks to calcium imaging techniques (Schrödel et al., 2013) that allow simultaneous recording of activity in a large fraction of neurons including the head ganglia (Kato et al., 2015) and also the ventral nerve cord and tail ganglia (Kaplan et al., 2020). These seminal works permitted an exploration beyond isolated sensory to motor pathways, allowing for whole brain recordings that reveal many overlapping pathways embedded in the recurrent wiring of the connectome. The experiments in Kato et al. (2015) showed the presence of well defined clusters with synchronized activity in the neural dynamics. By simultaneously registering the locomotor behavior of freely moving worms using automatic video tracking, they showed that these synchronized clusters can be correlated with action sequences of the nematode, suggesting that behaviorally relevant neural representations occur through the coordination of neuronal activity patterns at the level of global dynamics (Kato et al., 2015). Also, experimental results in Kaplan et al. (2020) showed that the global neural dynamics present a hierarchical structure across brain and motor circuits, where slower dynamics constrain the state and function of faster ones. Expanding the registration of the activity of the worm to also include head and body postures they showed that this hierarchy is used to coordinate behaviors across different time scales (Kaplan et al., 2020).

These experiments showed that organization of behavior in the worm is encoded in a hierarchical structure of globally distributed, continuous, and low-dimensional neural dynamics, leading to the conclusion that the behavioral states are encoded in the brain as an internal representation that emerges from the neurons and their circuit interactions. These results highlight the importance of studying the nervous system, the body and the environment as a coupled system, in order to understand the properties that emerge from their continuous dynamical interaction (Chiel and Beer, 1997; Clark, 1998; Webb, 2000, 2002; Floreano et al., 2014). In this context the use of robots appears as an attractive modeling tool, since it is possible to access and have full control over the parameters and dynamical variables that govern their behavior (Pfeifer et al., 2007; Izquierdo and Beer, 2013).

Also, the physical implementation of a robot allows for testing the performance of algorithms in a body that is subject to the laws of physics and is immersed in real time in a natural environment, thus avoiding the complex technicalities of simulating the environment, a procedure that involves its own models and therefore adds more variables, assumptions and noise to the analysis. In turn, this allows us to focus directly on the global dynamics that emerge from the connectome and how it affects behavior.

In this work we use a robotic vehicle that is controlled by a neural network numerical simulation based on the *C. elegans* connectome (Busbice, 2014b). In the simulation the neurons have the same firing threshold, and as a consequence have identical dynamics as isolated units. In this way the changes observed in their dynamics reflects their interactions through the non-uniform distribution of synaptic connectivity. This allows us to analyze the neural dynamics that emerges through the connectome, and at the same time to study the interplay between the neural dynamics and the actions of the robot.This provides a methodology to check if circuit interactions are enough to explain behavior or further assumptions are needed (Jabr, 2012; Morgan and Lichtman, 2013).

We find that some basic features of the global neural dynamics of the worm, such as the presence of clusters of synchronized neurons (Kato et al., 2015), and a nested hierarchical structure that couples slow and fast oscillating neurons (Kaplan et al., 2020), can be explained just as an emergent consequence of the connectome architecture, without need of any other modulatory mechanism.

## 2. The robotic model

In our experiments we use a robot design that essentially consists of a vehicle with two lateral motors connected to wheels and a distance sensor in the front [Supplementary Figure S1 (Industries, 2020b)]. This allows the vehicle to sense the environment and move on the ground. The neural simulation that controls the robot has elementary dynamical units that represent the neural dynamics, and uses the biological information of the connectome for their interaction. With this stripped down approach Busbice (2014a,b) showed that the robot presents emergent behaviors, which allow it to spontaneously navigate when sensory neurons associated with the presence of food are stimulated and to back up when confronted with an obstacle and sensory neurons associated to backing response are stimulated (see Supplementary Video S1). At the same time, the experimental results of Kato et al. (2015) show that the network dynamics interfaces with sensory neurons as early as the first synapse, providing a robust scaffold for sensory inputs to modulate behavior. Thus, the robotic model constitutes an excellent platform to study the interplay between connectome, neural dynamics and emergent behaviors. However, up to now neither the global dynamics of the model, nor its correlation with the underlying connectome and the emergent actions of the robot has been analyzed. In this work we fill this gap, as well as contrast, when possible, the results obtained using the robot with those coming from recent experiments on *C. elegans*.

We used two vehicles for our experiments. One of the robots is the commercially available GoPiGo robot by Dexter Industries (Industries, 2020b). The software that controls this robot is open source and can be downloaded from Industries (2020a). This allows for a straightforward reproduction of our experiments. Since the hardware of the GoPiGO robot is not open, we decided to build a custom robot with a similar design that can easily be constructed with off the shelf components at a low cost. In this way we provide an open hardware and open software alternative to reproduce the experiments (see Supplementary material for details). The numerical simulation was run in a Raspberry Pi (Foundation, 2020) computer mounted in the vehicles, that is also interfaced with the distance sensor and the motor control boards.

The control of the robots was implemented in a custom numerical simulation using the Python 3 programming language, and is based on the original program developed by Busbice (2014b) and the version implemented in the GoPiGo robot (Industries, 2020a).

In order to allow the robot to run in real time the program registers the neural states and then executes the output commands at every time step. The firing threshold of the neurons sets the time scale to the neural states, and the motor control program uses a single parameter to set the time scale for the motors to execute the output commands. In this way the program allows for the whole set of neural signals and the actions of the robot to be sampled once per second, setting the upper bound to the frequency of every individual signal to be ω = 0.5*Hz*. This is directly related to the physical scale of the robot and how fast it can execute the commands received (Busbice, 2014b). If the parameter for the motor control is set too low, then the motors cannot execute the actions, as they have a physical limitation on the time scale at which they can turn and reverse. If this parameter is set too high the robot actions take too long and there are no emergent behaviors to be observed in real time.

The microscopic dynamical units of the model, the neurons, evolve in a numerical simulation as oscillators. Our choice of single neurons as oscillators is based on the experimental evidence of Kato et al. (2015) and Kaplan et al. (2020). These experimental works highlight the oscillatory character of a number of *C. elegans* neurons. In fact, using principal component analysis Kato et al. (2015) show that the neural state's time evolution is cyclical, and that a large percentage of the full dataset variance can be accounted for by these neurons. Advancing a step forward, Kaplan et al. (2020) show that these oscillations present a hierarchical structure, where nested neuronal dynamics at different frequencies allow for multi-timescale behavioral organization.

The neurons evolve in the numerical simulation with discrete time steps. At each time step all the neurons add their input signals up to a given threshold value (*h* = 30). When a neuron *S*_*j*_ surpasses the threshold it fires, distributing the signal to its *S*_*i*_ neighbors, and resetting its state to zero. The neurons are updated in the following way:


(1) $$S_{i}(t+1)=\{\begin{array}{ll} S_{i}(t)+\sum_{j}W_{ij}\Theta \left[S_{j}(t)-h\right] & if\;S_{i}(t)\leq h\\ 0 & otherwise \end{array}$$

where *W*_*ij*_ is the synaptic weight between neurons *S*_*i*_ and *S*_*j*_, given by the connectome, and Θ is a step function.

If the threshold *h* is too low most of the neurons fire at every time step, and there are no emergent behaviors. If the threshold is too high, then almost no neuron fires, since many time steps are required for the neurons to reach the threshold. However, we observed that this value does not have to be precisely tuned, and obtained the same qualitative results for thresholds ranging between 10 and 100.

The neural network controlling the robot is based on the *C. elegans* connectome, allowing for the construction of a directed graph, where two connections to the same neuron represent a synaptic junction and a gap junction (Busbice, 2014b). The neurons in the nervous system of the worm can be divided into three categories according to their neuronal structural and functional properties: sensory neurons, interneurons and motor neurons (White et al., 1986; Varshney et al., 2011). According to a standard nomenclature every neuron has a name, that consists of either two or three uppercase letters indicating class, and corresponding number within a given class. If the neurons are radially symmetrical, each cell has a three-letter name followed by L (left), R (right), D (dorsal), or V (ventral). A complete list of *C. elegans* neurons, their lineage, and descriptions can be found in the WormAtlas database (Altun et al., 2002–2021; Altun, 2021).

In the robot, a laser distance sensor allows for very accurate distance measures, with a range up to two meters with a millimeter resolution. This sensor activates a number of sensory neurons associated with avoidance behavior (WormAtlas, 2021) when the distance from the robot to an obstacle is below a given threshold (30 cm).

The outputs of motor neurons connected to the left and right muscles of the worm involve both positive and negative weights, representing excitatory and inhibitory connections. They are accumulated into a value of either left or right, and this value is then used to control the rotation of the two wheels (Busbice, 2014b; Industries, 2020a). For a complete list of all the neurons involved in the sensory inputs and kinetic outputs see Supplementary data section in the Supplementary material. With this setup we conducted experiments where the robot was allowed to roam freely in a room with random obstacles, simultaneously recording the individual dynamics of all the neurons and also the actions of the robot.

We chose this particular design for our experiments due to its attractive features. On the one hand, the design of the robot allows for a straightforward construction that can be easily reproduced with low cost. On the other hand, the neural simulation that controls the robot has elementary dynamical units, and uses the biological information of the connectome for their interaction. With this stripped down approach Busbice (2014a,b) showed that the robot presents emergent behaviors, which allow it to spontaneously navigate and avoid obstacles (see Supplementary Videos S1, S2). Thus, the robotic model constitutes an excellent platform to study the interplay between the connectome and emergent behaviors. However, up to now neither the dynamics of the model, nor its correlation with the actions of the robot has been analyzed. The main objective of the present work is to fill that gap, as well as to contrast, when possible, the results obtained in the robot with those coming from recent experiments on *C. elegans*.

## 3. Results

### Emergent neural dynamics

#### Frequency and phase synchronization

**Figure 1:**
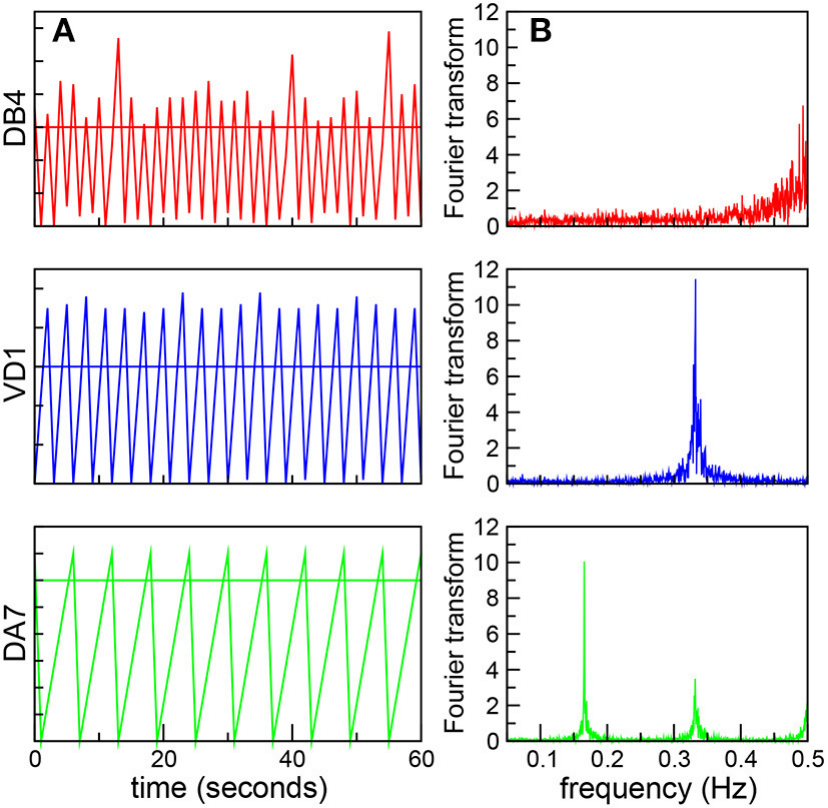
Neurons oscillate at different frequencies. **(A)** Dynamics of three ventral cord motor neurons, DB4 (top, red), VD1 (middle, blue), and DA7 (bottom, green). The figure shows the neural signals as a function of time in the same 60 s time interval. The horizontal lines shows the threshold value *h* = 30 **(B)** Corresponding Fourier transforms with sharp peaks, used to define the characteristic frequency Ω.

We will begin by analyzing the correlation between the global dynamics and the underlying connectome. It is worth stressing that in the robotic model all the neurons have the same firing threshold, and thus have identical individual dynamics as isolated units. However, we expect their dynamics to reflect the non-uniform distribution of synaptic connectivity. For example, neurons in a central position, with a large number of inputs or receiving connections with large weights can reach the threshold faster, and thus fire frequently. In fact, we found that AVAL, AVBR, and AVBL, nodes with the greatest degree centrality, and also with the largest in-closeness centrality (Varshney et al., 2011) oscillate with high frequency. In contrast, nodes in a peripheral position receive fewer inputs, and thus will take longer to reach the threshold, leading to a slower dynamics. In Figure 1A we plot the signals of three ventral cord motor neurons in a fixed time interval showing oscillations at different frequencies. We quantitatively characterize these oscillations by performing a Fourier transform (FT; Figure 1B). We find that the real value of the FT present sharp peaks, and thus define the characteristic frequency of the neurons, Ω, as the highest peak in the FT.

**Figure 2:**
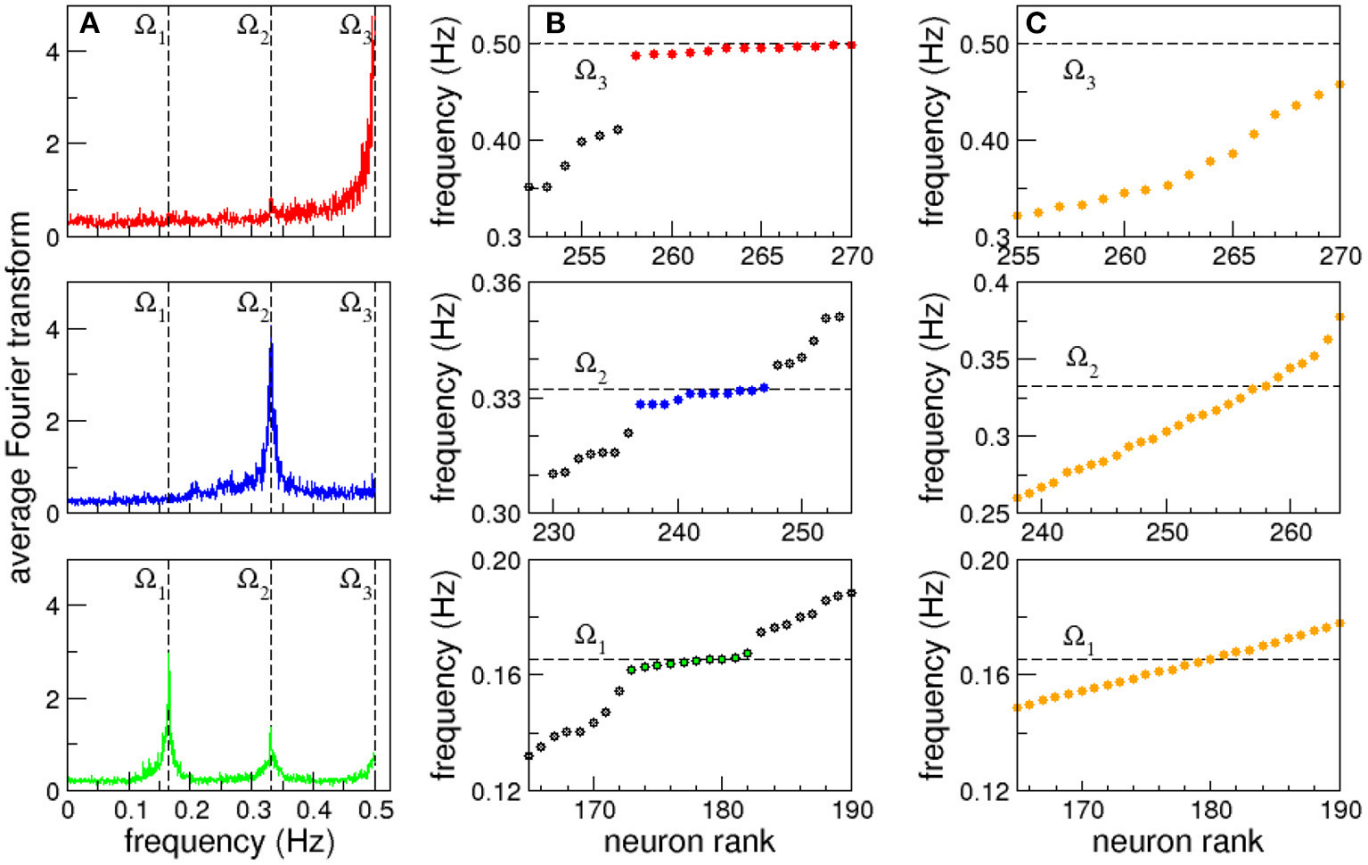
Frequency synchronization. **(A)** Average Fourier transforms of neurons in three synchronized clusters for a full 15 min long experiment. **(B)** Neurons sorted from lower to higher characteristic frequency. Dashed lines are shown as a guide to the eye to show the characteristic frequency of the synchronized clusters that appear as horizontal lines. **(C)** Synchronized clusters are not observed in control experiments with randomized versions of the connectome.

Clusters of neurons that present coordinated dynamics have been registered experimentally in *C. elegans* by Kato et al. (2015). With this idea in mind when we extend our focus from individual to collective dynamics we observe that some neurons are clustered in groups that share the same characteristic frequency. Even more, in some cases the complete shape of their FT, including smaller peaks, overlap. This allows us to define synchronized clusters as groups of neurons with overlapping FT. In Figure 2A we plot the average Fourier Transform of neurons in three different frequency synchronized clusters. The averaged Fourier transforms have sharp peaks, that allows us to define the clusters Ω by their characteristic frequency (mean ± standard deviation): Ω_1_ = 0.165 ± 0.002 Hz (green, bottom), Ω_2_ = 0.332 ± 0.003 Hz (blue, middle) and Ω_3_ = 0.496 ± 0.004 Hz (red, top; see also Supplementary Tables S1A–C and Supplementary Figures S2A–C for a complete list of neurons in each cluster, their individual signals and corresponding FTs). Notice that Ω_2_ and Ω_3_ are approximately integer multiples of Ω_1_. While the origin of such apparent harmonic relation remains unclear, having only three frequencies a random coincidence cannot be excluded. Figure 2B shows in detail the three synchronized clusters when the neurons are sorted from lower to higher characteristic frequency. We use dashed lines as a guide to the eye to show the characteristic frequencies Ω of the synchronized clusters that appear as horizontal lines. At this point it is worth stressing that the emergence of synchronized clusters does not seem to depend on the particular topography where the robot moves. This is due to the fact that the sensory neurons associated with avoidance behavior are only stimulated when the distance sensor measures a distance below the threshold, and given that then the robot responds by backing up, the time interval in which the sensory neurons are stimulated is usually very short. As a consequence, the global neural dynamics quickly converges again to a global attractor.

Figure 2B strongly resembles the formation of synchronized clusters in Kuramoto oscillators with a broad distribution of natural frequencies (Manrubia et al., 2004). In this case however, the nodes have identical dynamics, as all the neurons have the same firing threshold, and the heterogeneity arises from the complex network of interactions given by the *C. elegans* connectome. A direct comparison of the list of sensory inputs and kinetic outputs with the neurons in the synchronized clusters reveals that it is not a trivial effect of direct stimulation (see Supplementary data section in the Supplementary material). To test that the emergence of synchronized clusters is not an epiphenomenon, we analyzed the dynamics in randomized versions of the connectome where the degree distribution and weight distributions are conserved. That is, the random networks have exactly the same in and out degree and weight distributions randomly assigned to the nodes and links (Milo et al., 2002; Azulay et al., 2016). In all cases we found that the emergent actions of the robot are lost (see Supplementary Video S2), and no synchronized clusters are observed (see Figure 2C).

Kato et al. (2015) used calcium imaging techniques to record neural activity with single cell resolution in all the head ganglia and some ventral cord motor neurons. In these experiments ~ 100 neurons were scanned three times per second in 20 min long runs, generating high dimensional datasets. Using principal component analysis (Jolliffe and Cadima, 2016) for dimensional reduction they were able to cluster neurons with correlated signals. In particular, they produced neuron weight vectors (PCs), showing that clusters with opposite signs in their PCs oscillate in antiphase, and according to their weight vector sign are correlated with either forward or backward behavior of the worm.

**Figure 3:**
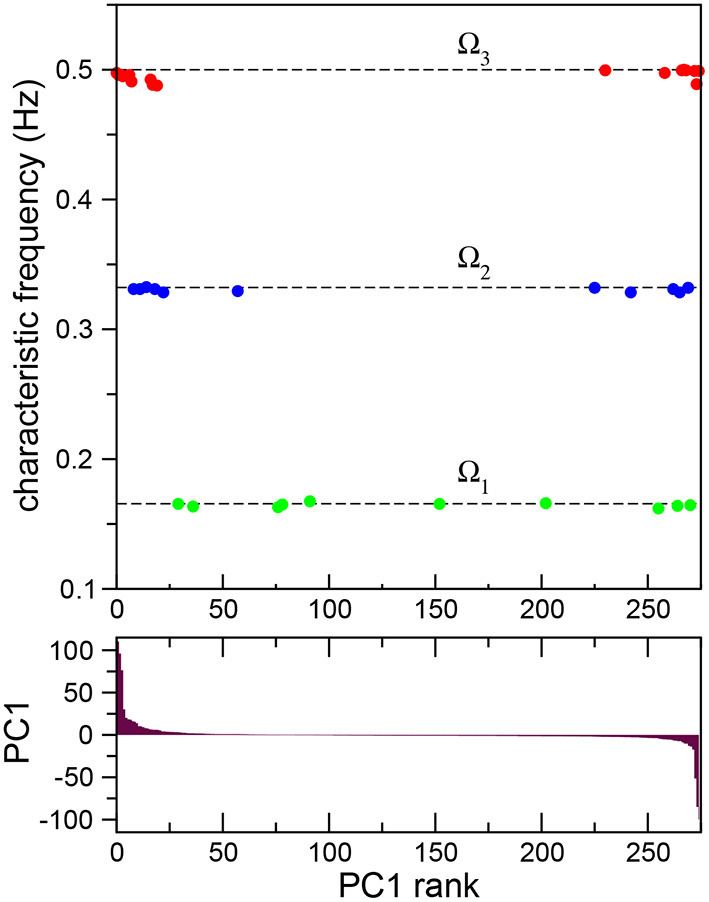
Frequency synchronization sorted according to principal component analysis. Average Fourier transforms of neurons in three synchronized clusters **(top)** for a full 15 min long experiment sorted according to their first principal component PC1 **(bottom)**. The analysis reveals the presence of subclusters with opposite signs in their principal component weight.

Following the same procedure we also performed a similar principal component analysis. As in Kato et al. (2015), each of the neural time series was normalized using the mean value ($\bar{s}$) and the standard deviation (σ_*s*_):


(2) $$s^{\prime }(t)=(s(t)-\bar{s}(t))/\sigma_{s}(t)$$

so that the new time series have now zero mean and unit standard deviation. Then, principal components were calculated based on the covariance structure of the normalized data (Kato et al., 2015; Jolliffe and Cadima, 2016), producing neuron weight vectors (PCs) for the neural time series of all the neurons in the robot. This allowed us to advance further in the quantitative characterization of the frequency synchronized clusters. In Figure 3 (top) we plot the characteristic frequencies the three frequency synchronized clusters Ω_1_ (green), Ω_2_ (blue), and Ω_3_ (red) already presented in Figure 2B. The neurons have now been sorted according to their first principal component weight (PC1; Figure 3 (bottom), i.e., the leftmost neurons have the highest positive value, while the rightmost have the lowest negative PC1. Note that all the synchronized clusters involve neurons including both positive and negative PC1 values. The cluster with lower frequency, Ω_1_, presents a broad distribution of neuron weights. For higher frequencies the neurons present a stronger segregation toward extreme PC1 values. In fact, both Ω_2_ and Ω_3_ are clearly divided into two smaller subclusters: one with only positive and another with only negative PC1 values. This segregation reflects differences in the firing times of the neurons. In each cluster the firing times of the neurons are proximal. In contrast, when one compares the signals of neurons between clusters a shift in their relative phase is observed. The largest shift occurs for extreme PC values, when the neurons in the different subclusters of the same frequency oscillate mostly in antiphase (see Supplementary Figure S8).

#### Nested neuronal dynamics

We analyze now how the collective oscillations in the frequency synchronized clusters are also coupled between themselves. In Figure 2A we show the average Fourier transforms of neurons in three frequency synchronized clusters. Note that the average FT of the cluster with the lowest frequency, Ω_1_, also has two smaller peaks, that coincide with the characteristic frequency of the other clusters: Ω_2_ and Ω_3_. As a guide to the eye we indicate with dashed lines these frequencies in the three panels. This reveals a coupling between the different frequency synchronized clusters.

**Figure 4:**
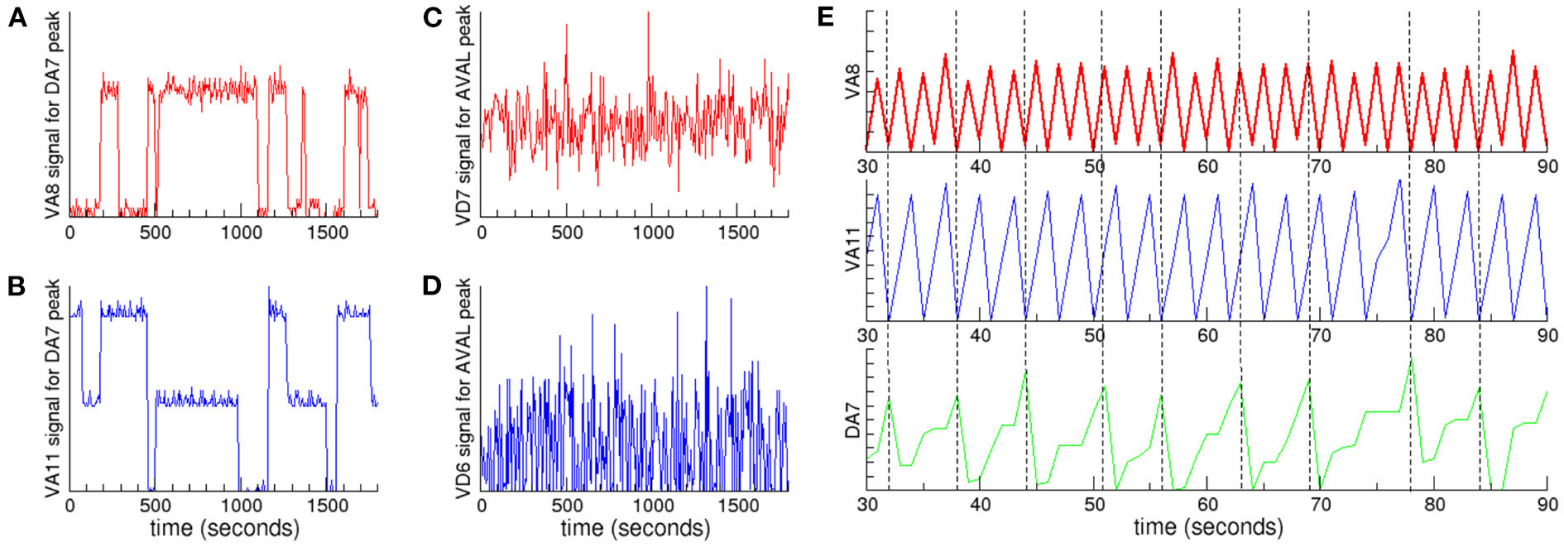
Nested neuronal dynamics. We plot the signals of neurons in clusters Ω_2_ and Ω_3_ when a neuron selected from cluster Ω_1_ reaches its maximum: **(A)** VA8 signal (red) from cluster Ω_3_ and **(B)** VA11 (blue) from cluster Ω_2_, when DA7 from cluster Ω_1_ reaches its maximum. Coupling between neurons in these clusters appears as regions characterized by signals with small fluctuations around a mean value. In contrast, when there is no coupling the signals present large fluctuations, as shown for example for the signals of **(C)** VD7 (red) from cluster Ω_3_ and **(D)** VD6 (blue) from cluster Ω_2_, when AVAL from cluster Ω_1_ reaches its maximum. **(E)** A 60 s time interval from the data presented in **(A, B)** shows the hierarchical relation. The signals correspond to VA8 (red), VA11 (blue) and DA7 (green). The vertical dashed lines are a guide to the eye showing the time when DA7 reaches its maximum.

In order to quantitatively study the coupling between oscillations at different frequencies, we analyzed the signals of neurons in a given cluster when the signal of a neuron in another cluster with a lower characteristic frequency reaches its maximum (Jensen and Colgin, 2007). If there is a coupling between the oscillations, then we expect that the signal of the neuron with highest frequency could present small fluctuations around the same mean value every time the maximum with lowest frequency is reached. On the contrary, if there is no coupling, then the signal is expected to vary randomly. In Figure 4 we plot the signals of neurons in Ω_2_ and Ω_3_ when a neuron selected from the cluster with lower characteristic frequency Ω_1_ reaches its maximum. In particular, we focuse our attention in the neurons with the highest positive PC1 weight in each of the synchronized clusters. Figures 4A, B show that these neurons are coupled, as the same mean value persists in extended time intervals. Also, to visualize this result we plot in Figure 4E the signals of these neurons in a fixed time interval, using vertical dashed lines as a guide to the eye. The figure clearly shows a nested hierarchical relation between oscillations at different frequencies. In Figures 4C, D we plot the signals of the neurons that have the lowest negative PC1 weights. In sharp contrast to neurons with positive weights, the signals are not correlated, and fluctuate throughout the whole experiment.

### Neural dynamics and robot actions

In this section we analyze the correlation between the emergent neural dynamics and the actions of the robot. The robotic model is well suited for this study, since it allows for recording of neural dynamics while simultaneously registering actions. To determine if there was a correlation between a given action and the activity of specific neurons, we contrasted the time series of the neural dynamics with the time series of robot actions. First, we registered all the action events in a 20 min long experiment. Then, we analyzed which actions the robot was executing when a given neuron had fired, that is, when its value was reset to zero. Finally, we contrasted the fractions of events in this two time series to see if there were significant variations.

**Figure 5:**
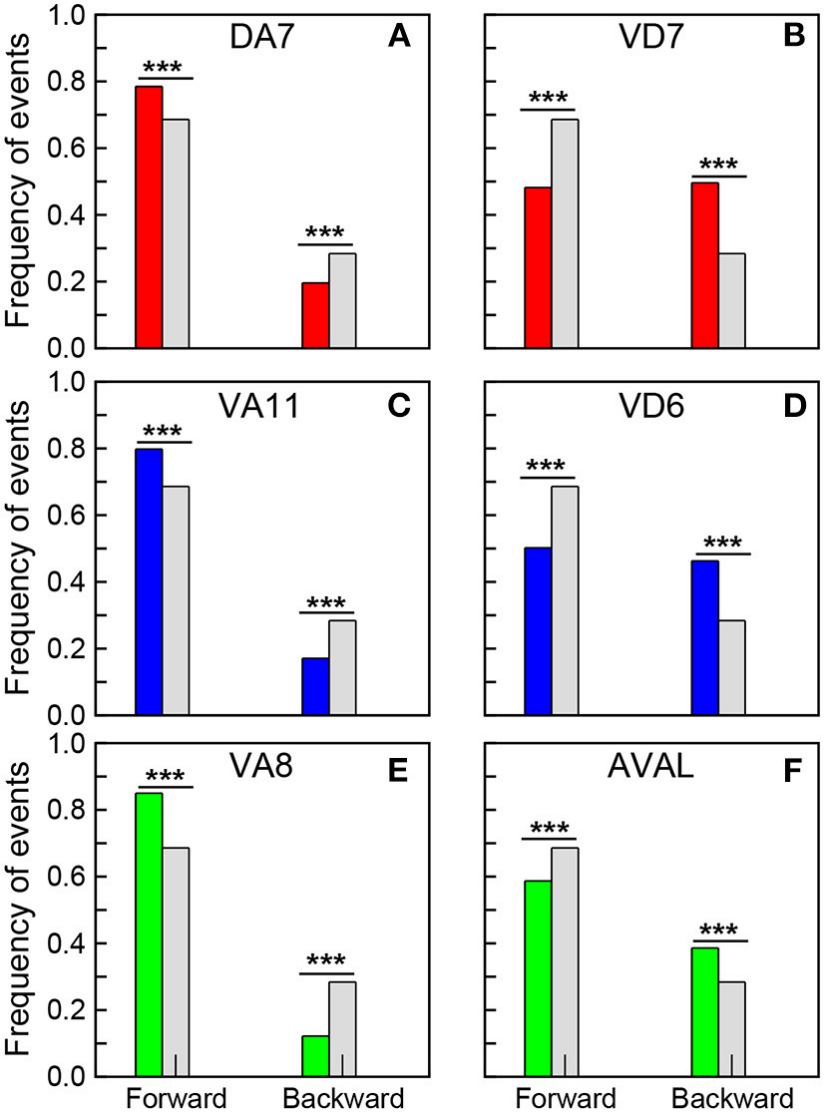
Correlation of neural dynamics with robot actions. Fraction of forward and backward events registered when the selected neurons fired. **(A)** DA7, **(C)** VA11, and **(E)** VA8 correspond to the neurons with the largest positive PC1 in the synchronized clusters Ω_1_, Ω_2_ and Ω_3_, while **(B)** VD7, **(D)** VD6, and **(F)** AVAL correspond to the neurons with the lowest negative PC1 in these clusters. The gray bars correspond to the fraction of forward and backward events in a 20 min long experiment. A chi-square test (statistically significant, ***p-value < 0.00001) show that the opposing effects of all these neurons in the actions of the robot are significant.

In the experiments the robot moves mostly forward and backward, while the turnings are usually short events where the robot only changes its direction, so we focus mainly on forward and backward events. These events correspond to the network response to the stimulation of sensory neurons (WormAtlas, 2021). As observed in the worm, the stimulation of chemotaxis neurons promotes sustained roaming, and makes the robot to move forward, while the stimulation of sensory neurons that promote avoidance makes the robot to move backwards (Busbice, 2014a,b). At this point it is worth stressing that these action responses are emergent and not trivial, in the sense that only a small fraction of all the dynamical units is stimulated, and thus the responses correspond to a modulation of the global dynamics. We found that the neurons in the synchronized clusters with the largest positive PC1 weights promote forward events, while the neurons with the lowest negative weights promote backward events. Figure 5 shows the fraction of events registered when the neurons with the largest positive and lowest negative PC1 weights in the synchronized clusters fired (see also Supplementary Table S2). The colored bars correspond to: Ω_1_ (green), Ω_2_ (blue), and Ω_3_ (red). In each figure, the results are contrasted with the fractions of events for the whole experiment (gray bars). Note that a significant increase in forward events is observed when the neurons with the largest positive PC1 fire (first columns in Figures 5A, C, E), while a reduction is observed in the neurons with the lowest negative PC1 (first columns in Figures 5B, D, F). At the same time, an increase in backward events is observed when neurons with negative PCs fire (dashed columns in Figures 5B, D, F), while a decrease in the fraction of backward events is observed in neurons with positive weights (dashed columns in Figures 5A, C, E). These results reveals the role that the segregation of the neurons in the synchronized clusters plays in promoting different actions to be executed by the robot. Interestingly, in *C. elegans*, Kato et al. (2015) noted that neurons that promote opposing behaviors, such as backward and forward crawling, also have opposing signs of their PC1 weights.

## 4. Discussion

The nematode *C. elegans* is a model organism that allows for an integrated view where the continuous interaction between the nervous system, the body and the environment can be studied as a coupled dynamical system. In two seminal works Kato et al. (2015) and Kaplan et al. (2020) showed that organization of behavior in the worm is encoded in a hierarchical structure of globally distributed, continuous, and low-dimensional neural dynamics. These results led to the conclusion that the behavioral states are encoded in the brain as an internal representation that emerges from the neurons and their circuit interactions. Our analysis of the neural dynamics and its correlation with the actions in a robotic model based on the connectome of the nematode *C. elegans* allowed us to test this hypothesis, at least as a first approximation. At this point it is worth stressing that our goal was not to establish a one-to-one comparison between the robotic vehicle and the worm. In fact, we chose to build robots following the idea proposed by Busbice due to its stripped down design, that allows for a complex system approach, where the interaction of extremely simple dynamical units through the complex network defined by the connectome allows for the emergence of a global dynamics. With this approach we have shown a number of emergent characteristics which are also observed in the worm, such as the emergence of synchronized clusters of neural activity that can be correlated with actions.

Surprisingly, we also observed the emergence of a nested hierarchical structure. In the worm, Kaplan et al. (2020) showed that the presence of a hierarchical structure in the neural dynamics allows for the coordination of behaviors across different time scales. This include head movements, body undulations, and also forward and reverse bouts. It is worth stressing that the simple design of the robot we use does not allow for the execution of some of this behaviors. Nevertheless, Kaplan et al. (2020) observed that the hierarchical structure persisted even when the animals were immobilized, concluding that it is an intrinsic property that emerges from the neurons and their circuit interactions. The results obtained with the robot highlight this conclusion, i. e. that the presence of a nested dynamics is an emergent property of the interactions of the neurons through the connectome.

Summarizing, we have shown that a number of characteristics observed in the neural dynamics of *C. elegans* can be attributed to the complex network structure of the connectome. We expect that some of our conclusions can be extended to other connectomes, as a number of common principles have been identified in the comparison of the topological layout of nervous systems across species (Van den Heuvel et al., 2016). At the same time, differences in the results can shed light in understanding the effects of variations that are species specific. As larger neural networks and ever increasing detail on their dynamics are registered, the complexity and high dimensionality of the data will set new challenges for analysis and interpretation. For example the Drosophila hemibrain connectome involves ~ 25,000 neurons, including regions involved in functions such as associative learning, fly navigation and sleep (Scheffer et al., 2020). Simple robotic models as the one we analyzed here can provide a useful tool to test behavioral hypothesis relating neural structure and function.

## Data availability statement

The datasets presented in this study can be found in online repositories. The names of the repository/repositories and accession number(s) can be found at: https://gitlab.com/neuro-rob-tica/neuro-robotica-c-elegans.

## Author contributions

All authors contributed to manuscript revision, read, and approved the submitted version.
