# Supplementary material for: Emergent dynamics in a robotic model based on the Caenorhabditis elegans connectome
Source: Front Neurorobot. 2023 Jan 9;16:1041410. doi: 10.3389/fnbot.2022.1041410 (PMC9868850; doi:10.3389/fnbot.2022.1041410)
Supplement: Supplementary file 3 [file Data_Sheet_1.PDF]

frontiers

Supplementary Material

1 Supplementary Data

Every robot consists of a vehicle with two lateral motors connected to wheels and a distance sensor in the front. This allows the vehicles to sense the environment and move on the ground. Both robots use a laser distance sensor that allows for very accurate distance measurements, with a range up to two meters and millimetre resolution. The two robots are equivalent and the results obtained with both were essentially the same

Step-by-step instructions to build the GopiGo robot are available here:

https://gopigo.io/getting-started

and also, step-by-step instructions on how to connect the distance sensor to this robot are available here:

https://www.dexterindustries.com/GoPiGo/get-started-with-the-gopigo3-raspberry-pi-robot/4-attach-the-camera-and-distance-sensor-to-the-raspberry-pi-robot/.

The custom-made robot follows essentially the same building steps, using an L9110S dual motor driver instead of the GoPiGo 2 board for motor control. In the following two figures we show the wiring diagram for the custom robot. This includes the wiring to connect the distance sensor directly to the Raspberry Pi 3B and also the wiring of the L9110s motor driver.

Sensory Inputs and Kinetic Outputs

The robot follows the design proposed by Busbice, using the C. elegans connectome (available at https://github.com/openworm/) to control a two wheeled robot.

The neurons that are activated to promote spontaneous exploration are: [ADFL, ADFR, ASGR, ASGL, ASIL, ASIR, ASJR, ASJL]

For more details see

https://www.wormatlas.org/neurons/Individual%20Neurons/Neuronframeset.html

The neurons that are activated when the distance sensor measures a distance below the threshold are: [FLPR, FLPL, ASHL, ASHR, IL1VL, IL1VR, OLQDL, OLQDR, OLQVR, OLQVL]

For more details see

https://wormatlas.org/hermaphrodite/nervous/Images/neurotable1leg.htm

The motor neuron outputs to the following muscles are accumulated to control the left wheel:

Dorsal Muscles: [MDL07, MDL08, MDL09, MDL10, MDL11, MDL12, MDL13, MDL14, MDL15, MDL16, MDL17, MDL18, MDL19, MDL20, MDL21, MDL22, MDL23]

Ventral Muscles [MVL07, MVL08, MVL09, MVL10, MVL11, MVL12, MVL13, MVL14, MVL15, MVL16, MVL17, MVL18, MVL19, MVL20, MVL21, MVL22, MVL23]

while the motor neuron outputs to the following muscles are accumulated to control the right wheel:

Dorsal Muscles [MDR07, MDR08, MDR09, MDR10, MDR11, MDR12, MDR13, MDR14, MDR15, MDR16, MDR17, MDR18, MDR19, MDR20, MDL21, MDR22, MDR23]

Ventral Muscles [MVR07, MVR08, MVR09, MVR10, MVR11, MVR12, MVR13, MVR14, MVR15, MVR16, MVR17, MVR18, MVR19, MVR20, MVL21, MVR22, MVR23]

2 Supplementary Figures and Tables

2.1 Supplementary Figures

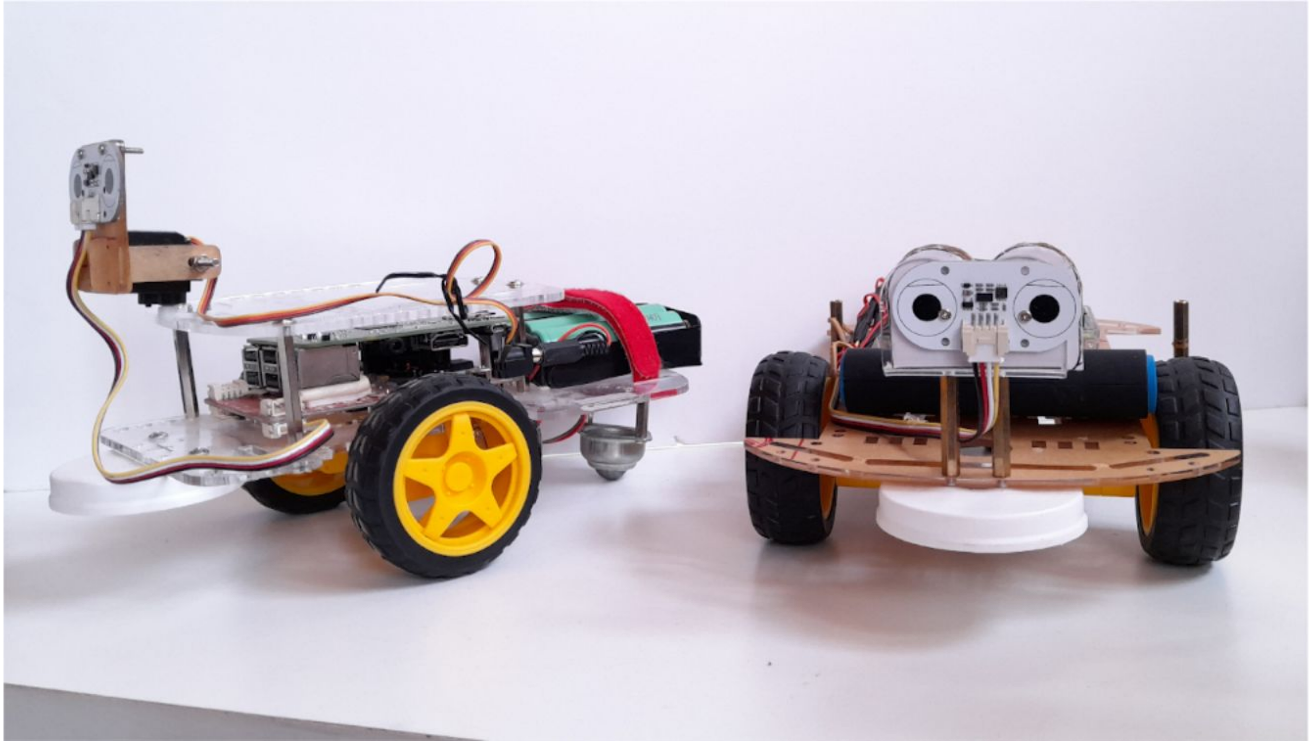

Supplementary Figure 1. The photo shows the two robots that we used in our experiments:

The robot on the left is a commercially available robot by Dexter Industries, called a GoPiGo robot https://www.dexterindustries.com/hacking-the-worm-brain-with-a-gopigo-and-raspberry-pi/

The software that controls this robot is open and can be downloaded from Github (https://github.com/Connectome/GoPiGo/blob/master/GoPiGoConnectome.py), however, the hardware of the GoPiGo robot is not open. For this reason, we decided to build a custom robot shown on the right. We used an L9110S dual motor driver instead of the GoPiGo 2 board for motor control. In this way the robot can easily be constructed with off the shelf components at a low cost.

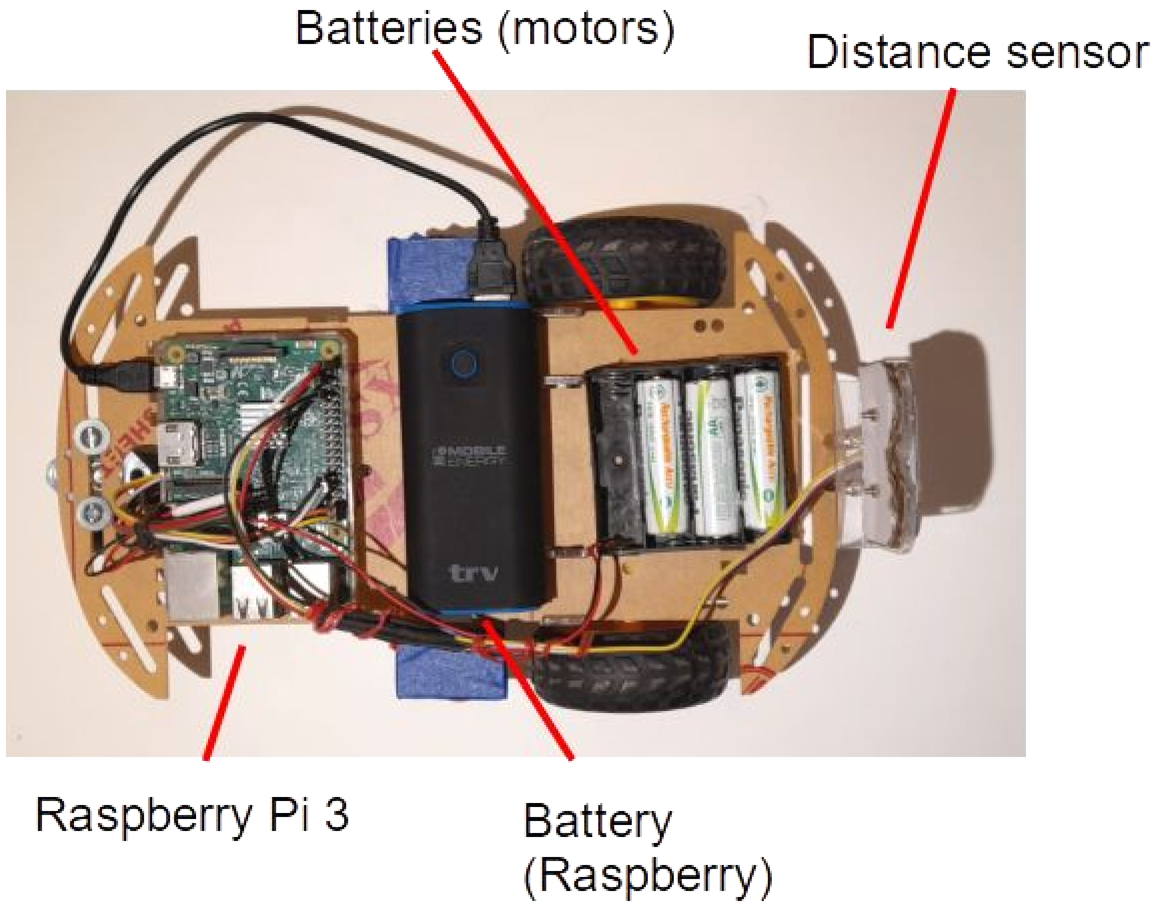

Supplementary Figure 2. Custom Robot view from above. The photo shows on the left the Raspberry Pi, where the numerical simulation that controls the robot runs. The rectangular black box is the battery that powers the Raspberry Pi. A separate battery pack is used to power the motors. A distance sensor is positioned on the front of the robot.

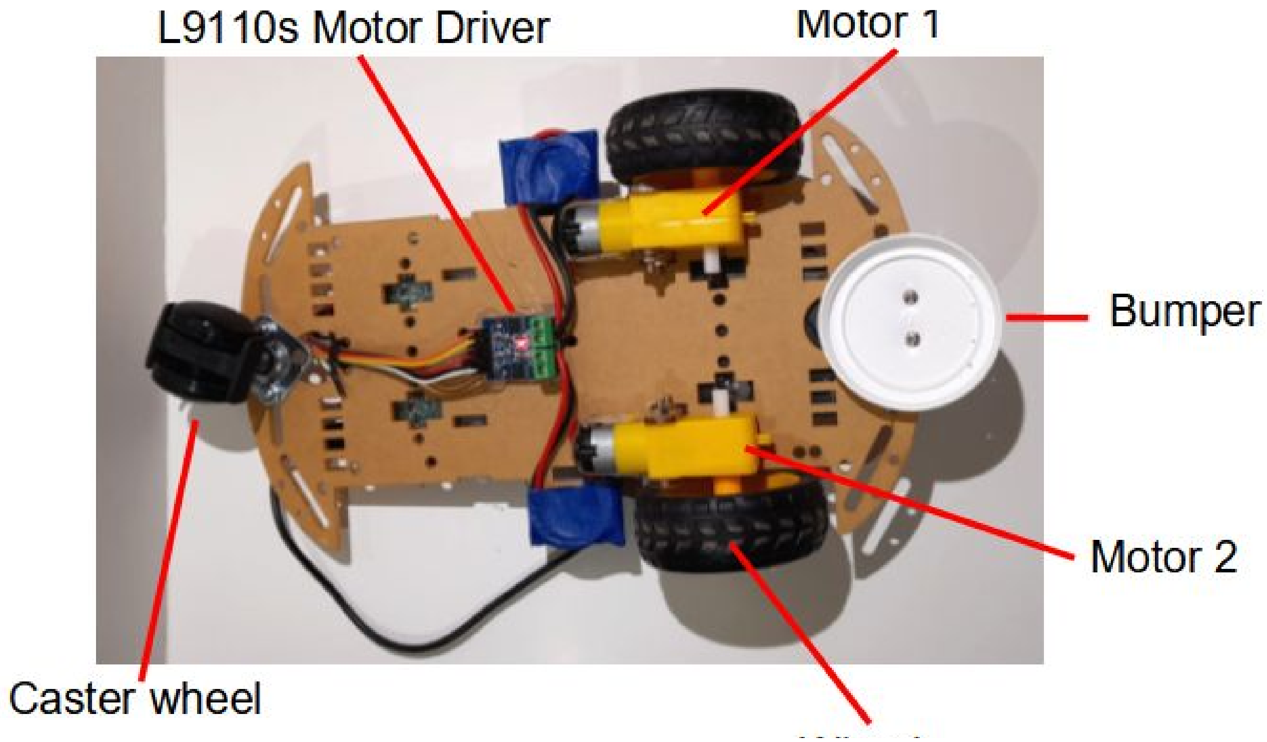

Supplementary Figure 3. Custom Robot view from below. A caster wheel on the left allows for support and robot maneuvering. The small L9110s motor driver is attached to the bottom, allowing for a direct connection to the motors (yellow), that control the wheels. A rubber bumper is attached to the front of the vehicle to protect the distance sensor from collisions.

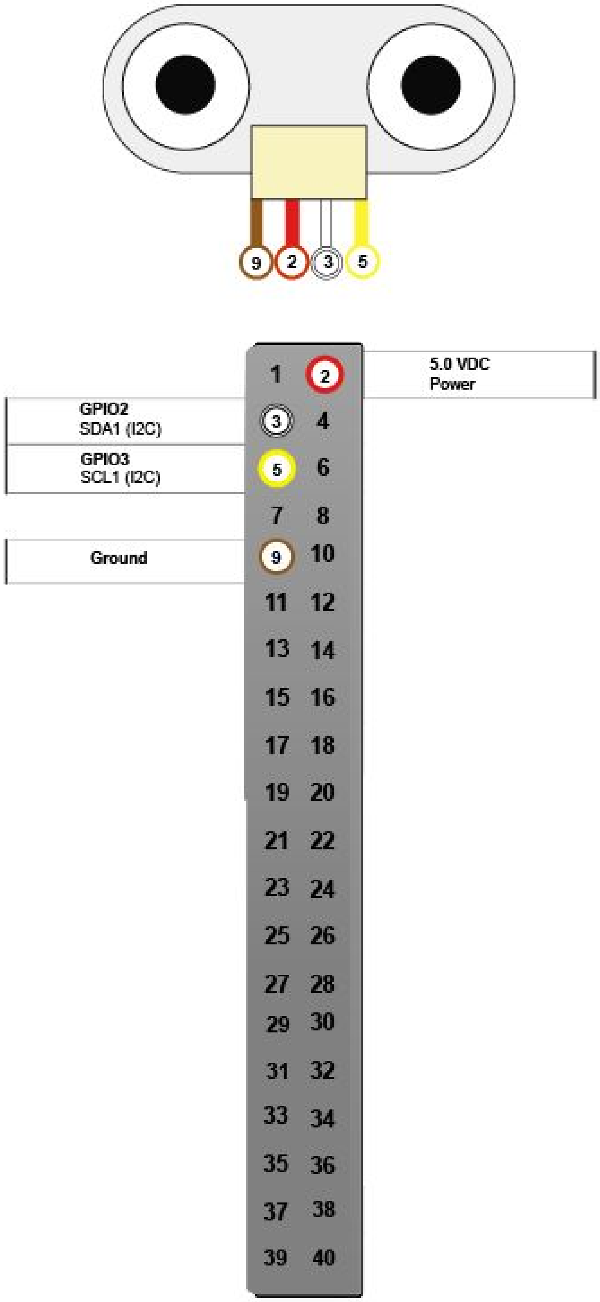

Supplementary Figure 4. Distance sensor wiring. The figure shows how the GOPIGO distance sensor is connected directly to the GPIO headers of a Raspberry Pi 3B in the custom robot.

The connections are: Ground, pin 9 (brown), power, pin 2 (red), SDA1 I2C, pin 3 (GPIO2, white) and SCL1 I2C, pin 5 (GPIO3, yellow).

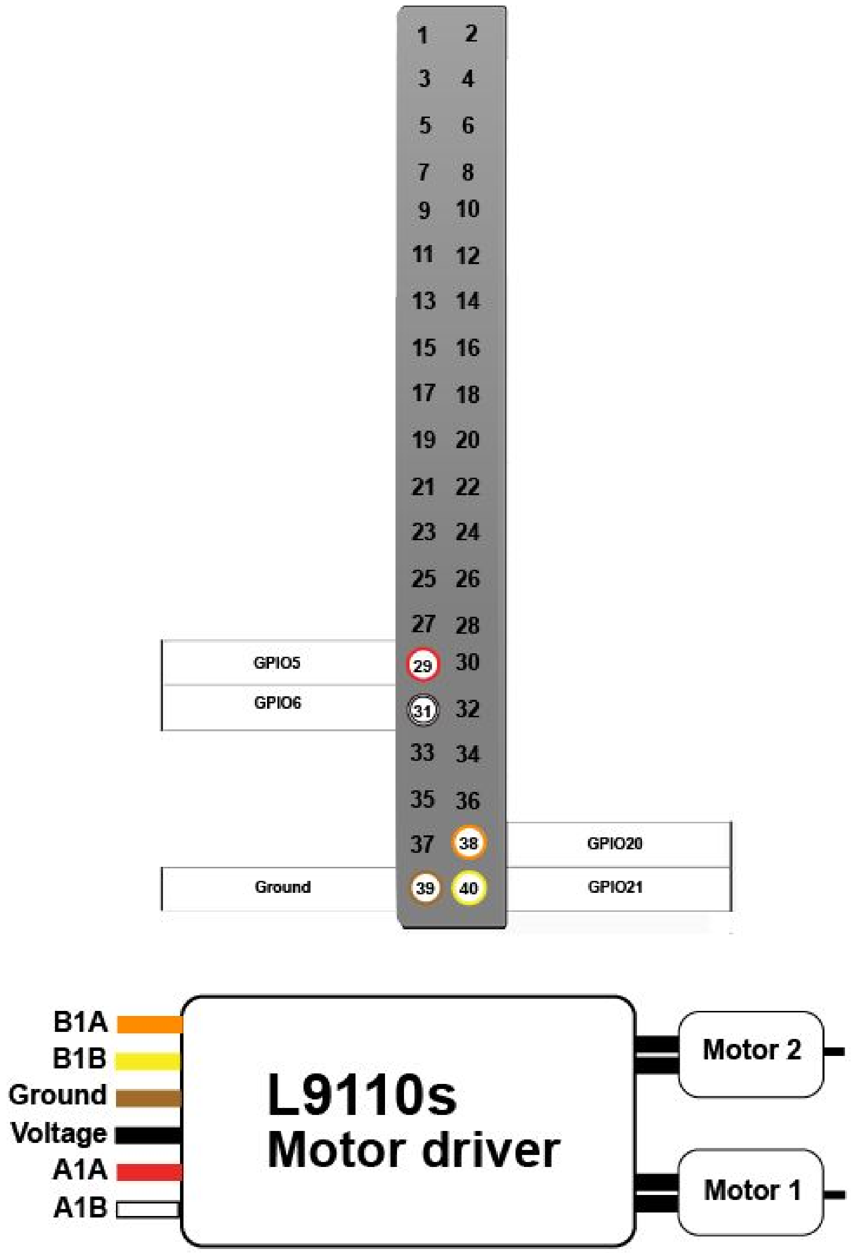

Supplementary Figure 5. Motor driver wiring. The figure shows how the motor driver L9110s is connected to the two motors and also to the GPIO headers of a Raspberry Pi 3B in the custom robot. The connections are: B1A, pin 38 (GPIO20, orange), B1B, pin 40 (GPIO21, yellow), Ground, pin 39 (brown), Voltage (direct conection to 5V battery, black), A1A, pin 29 (GPIO5, red) and A1B, pin31 (GPIO6, white).

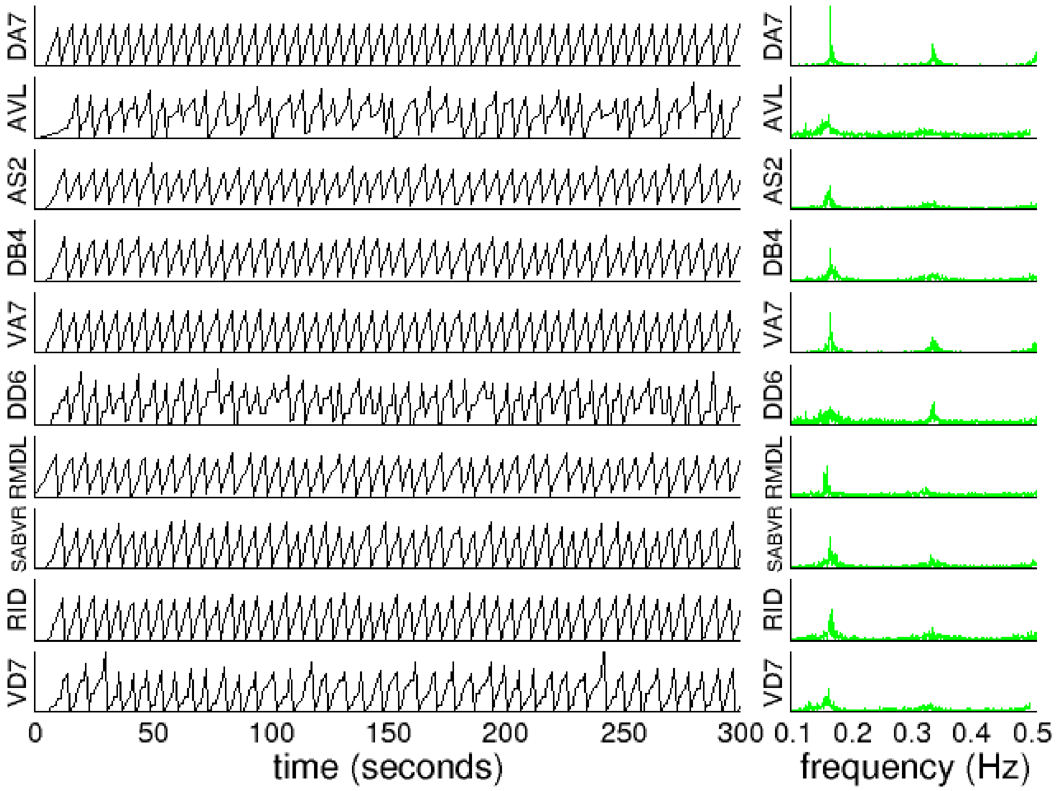

Supplementary Figure 6. The signals of all the neurons in the synchronized cluster Ω_1 in a five minute interval, and their corresponding Fourier transforms in a 20-minute-long experiment.

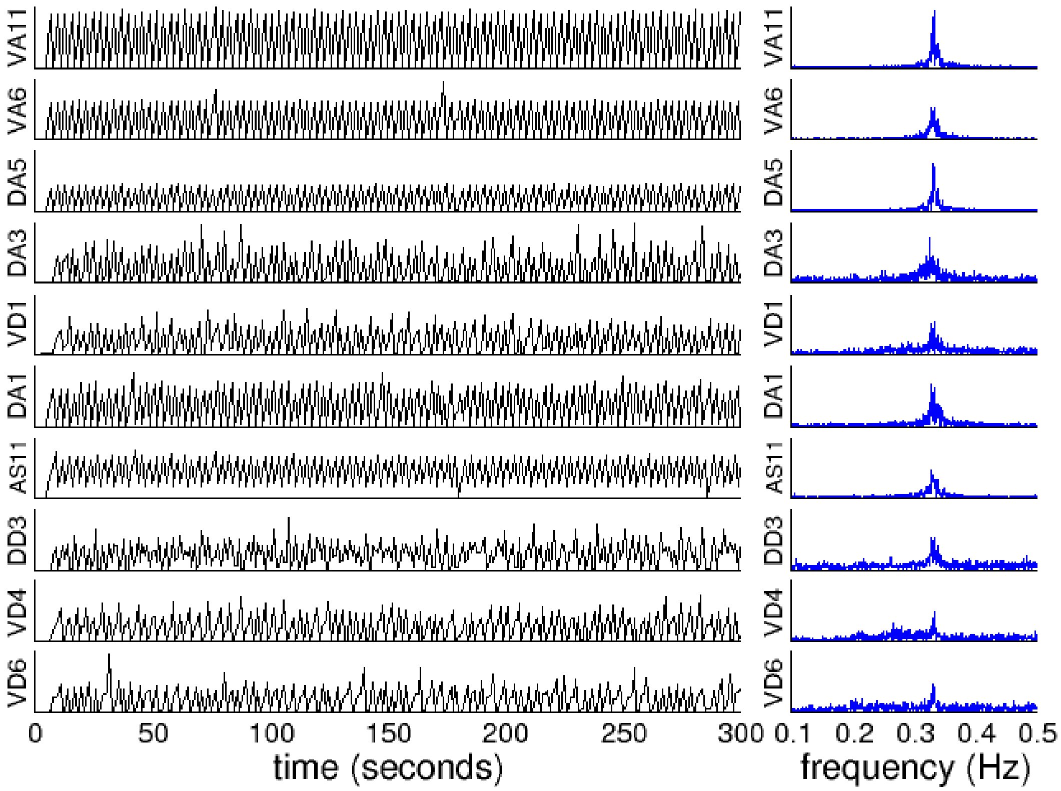

Supplementary Figure 7. The signals of all the neurons in the synchronized cluster Ω_2 in a five-minute time interval, and their corresponding Fourier transforms in a 20-minute-long experiment.

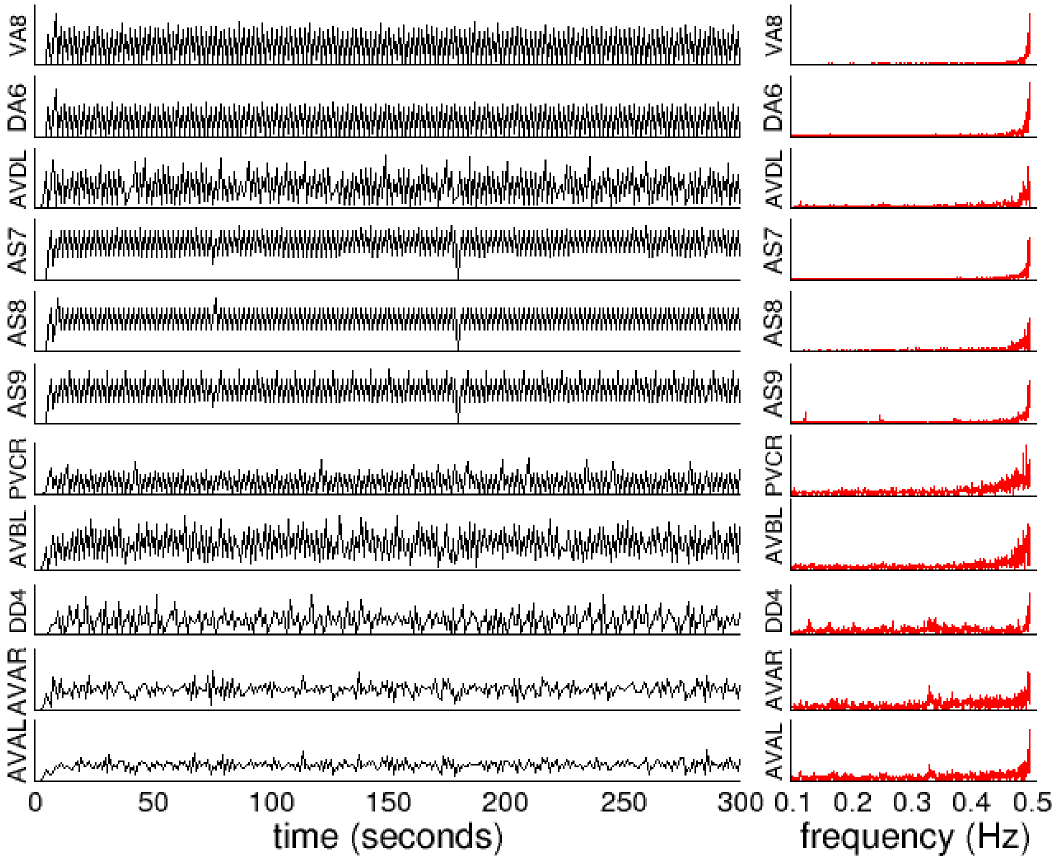

Supplementary Figure 8. The signals of all the neurons in the synchronized cluster Ω_3 in a five-minute time interval, and their corresponding Fourier transforms in a 20-minute-long experiment.

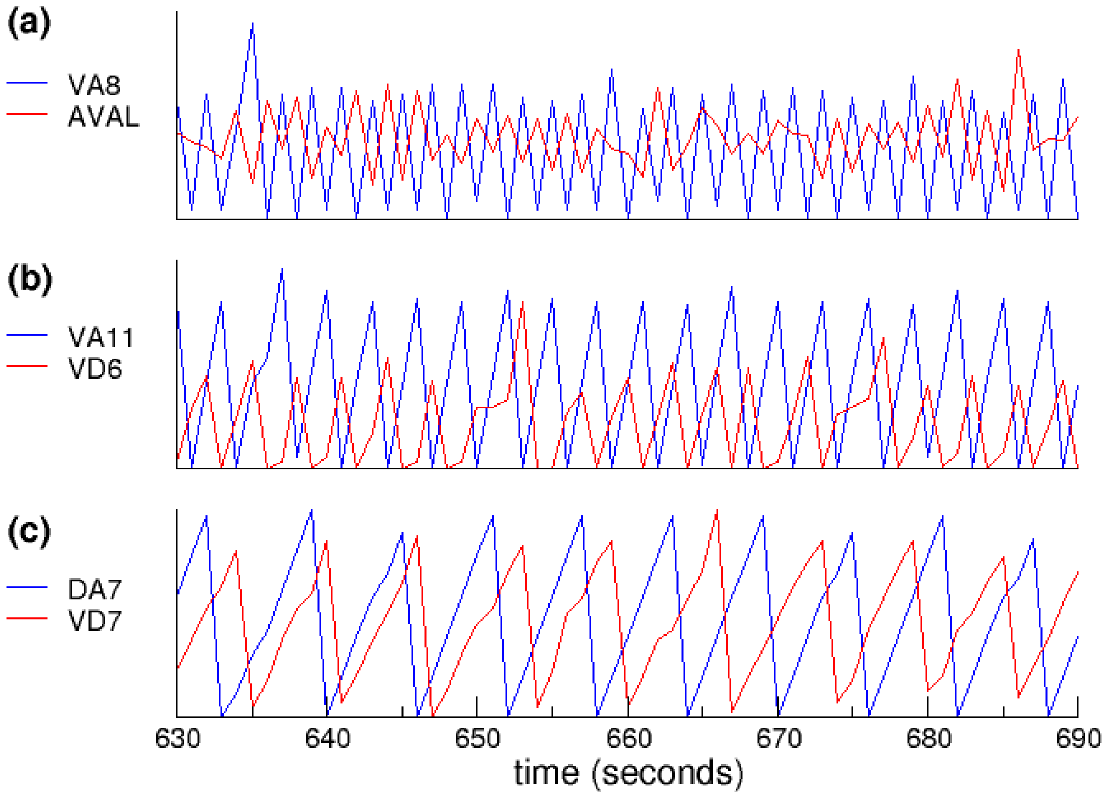

Supplementary Figure 9. Antiphase oscillations of neurons with the largest positive PC1 (blue) and lowest negative PC1 (red). (a) VA8 and AVAL in cluster Ω_3,(b) VA11 and VD6 in cluster Ω_2, and (c) DA7 and VD7 in cluster Ω_1.

All figures are presented in the same 60 second time interval.

2.2 Supplementary Tables

Neuron	Description	Ω	PC1
DA7	VCMN	0.166 ± 0.001	2.7 ± 0.1
AVL	RVCI	0.164 ± 0.001	1.6 ± 0.1
AS2	VCMN	0.163 ± 0.001	0.1 ± 0.1
DB4	VCMN	0.165 ± 0.001	0.1 ± 0.1
RMDL	VCMN	0.167 ± 0.001	-0.2 ± 0.1
VA7	VCMN	0.166 ± 0.001	-0.8 ± 0.1
DD6	VCMN	0.166 ± 0.001	-1.2 ± 0.1
SABVR	RI	0.162 ± 0.001	-4.2 ± 0.1
RID	RI	0.164 ± 0.001	-7.1 ± 0.1
VD7	VCMN	0.165 ± 0.001	-13.5 ± 0.1

Supplementary Table 1. The table presents the neurons in synchronized cluster Ω_1, their description and corresponding characteristic frequency and first principal component weight PC1. VCMN = Ventral Cord Motor Neuron, RVCI = Ring and Ventral Cord Interneuron, RI = Ring Interneuron.

Neuron	Description	Ω	PC1
VA11	VCMN	0.331 ± 0.001	15.3 ± 0.1
VA6	VCMN	0.331 ± 0.001	9.8 ± 0.1
DA5	VCMN	0.333 ± 0.001	7.4 ± 0.1
VD3	VCMN	0.328 ± 0.001	3.9 ± 0.1
VD1	VCMN	0.330 ± 0.001	0.6 ± 0.1
DA1	VCMN	0.332 ± 0.001	-1.7 ± 0.1
AS11	VCMN	0.328 ± 0.001	-2.5 ± 0.1
DD3	VCMN	0.331 ± 0.001	-6.4 ± 0.1
VD4	VCMN	0.328 ± 0.001	-7.1 ± 0.1
VD6	VCMN	0.332 ± 0.001	-12.7 ± 0.1

Supplementary Table 2. The table presents the neurons in synchronized cluster Ω_2, their description and corresponding characteristic frequency and first principal component weight PC1.

VCMN = Ventral Cord Motor Neuron.

Neuron	Description	Ω	PC1
VA8	VCMN	0.498 ± 0.001	110.1 ± 0.1
DA6	VCMN	0.496 ± 0.001	95.8 ± 0.1
AVDL	VCI	0.496 ± 0.001	76.1 ± 0.1
AS7	VCMN	0.495 ± 0.001	29.9 ± 0.1
AS8	VCMN	0.496 ± 0.001	17.8 ± 0.1
AS9	VCMN	0.491 ± 0.001	5.7 ± 0.1
PVCR	VCI	0.488 ± 0.001	15.1 ± 0.1
AVBL	VCI	0.499 ± 0.001	-10.1 ± 0.1
DD4	VCMN	0.499 ± 0.001	-51.2 ± 0.1
AVAR	VCI	0.489 ± 0.001	-84.9 ± 0.1
AVAL	VCI	0.499 ± 0.001	-99.8 ± 0.1

Supplementary Table 3. The table presents the neurons in synchronized cluster Ω_3, their description and corresponding characteristic frequency and first principal component weight PC1.

VCMN = Ventral Cord Motor Neuron, VCI = Ventral Cord Interneuron

Neuron	Forward	Backward
DA7	0.79	0.20
VD7	0.48	0.50
VA11	0.80	0.17
VD6	0.50	0.46
VA8	0.85	0.12
AVAL	0.59	0.39

Supplementary Table 4. Fraction of forward and backward events registered when the corresponding neurons fired. The fraction of events for the full 20-minute experiment was Forward = 0.69 and Backward = 0.28.
